# Multivitamin Supplementation and Fertility Outcome: A Retrospective Single-Center Cohort Study and the Clinical and Medicolegal Value of Nutritional Counseling

**DOI:** 10.3390/life15010048

**Published:** 2025-01-02

**Authors:** Giuseppe Gullo, Simona Zaami, Adriana Vita Streva, Sofia Burgio, Valentina Billone, Yulia Kotlik, Elena Chitoran, Silvia Ganduscio, Giovanni Baglio, Antonio Perino, Gaspare Cucinella

**Affiliations:** 1 Department of Obstetrics and Gynecology, Villa Sofia Cervello Hospital, University of Palermo, 90146 Palermo, Italy; gullogiuseppe@libero.it (G.G.); adriana.streva@gmail.com (A.V.S.); sofiaburgio4@gmail.com (S.B.); valentina.billone@gmail.com (V.B.); yulia.kotlik@gmail.com (Y.K.); gandusciosilvia@gmail.com (S.G.); antonio.perino@unipa.it (A.P.); gaspare.cucinella@unipa.it (G.C.); 2 Department of Anatomical, Histological, Forensic and Orthopedic Sciences, Sapienza University of Rome, 00161 Rome, Italy; 3 Surgery Department, “Carol Davila” University of Medicine and Pharmacy, 050474 Bucharest, Romania; chitoran.elena@gmail.com; 4 Italian National Agency for Regional Healthcare Services, 00187 Rome, Italy; baglio.giovanni@yahoo.it

**Keywords:** multivitamin supplementation, resveratrol, in vitro fertilization (IVF), intracytoplasmic sperm injection (ICSI), nutritional counseling

## Abstract

Resveratrol can beneficially affect growth and follicle development and lead to improved sperm function parameters in pre-clinical studies, while information from clinical studies is still inconclusive. This study aims to evaluate the biological and clinical impact of a resveratrol-based multivitamin supplement on level II assisted reproduction cycles (IVF and intracytoplasmic sperm injection [ICSI]). A retrospective, case-control study, involving 70 infertile couples undergoing IVF/ICSI cycles, was conducted at the Assisted Reproductive Center, Obstetrics and Gynecology Unit-Villa Sofia-Cervello Hospital in Palermo. The study group underwent pre-treatment with a daily nutraceutical based on resveratrol, whereas the control group received 400 mcg/day of folic acid. Primary endpoints to be evaluated were the number of mature follicles developed (>16 mm), total oocytes and Metaphase II (MII) oocytes retrieved, fertilization rate, number of embryos/blastocysts obtained, and semen quality. Secondary objectives in our evaluation were the duration and dosage of gonadotropins, the starting dose, the number of blastocysts to be transferred and frozen, implantation rate, and, ultimately, biochemical and clinical pregnancy rates. In the study group, a significantly higher number of mature follicles, oocytes, and MII oocytes were collected compared to the control group. In the study group, a higher fertilization rate as well as higher numbers of cleavage embryos per patient, blastocysts per patient, and frozen blastocysts were obtained. In the study group, a shorter administration time and lower dosages of gonadotropins required to reach follicle maturity were also observed compared to controls, with fewer dose adjustments during stimulation compared to the starting dose. No significant differences were found in biochemical or clinical pregnancy rates. A 12-month period of dietary supplementation with a resveratrol-based multivitamin nutraceutical leads to better biological effects on ICSI cycles.

## 1. Introduction

Nutrition has a huge impact on physical and mental health. It is through diet that humans can provide the body with the energy and nutrients needed to perform all its functions. It should therefore not be surprising that food can have a huge influence on reproductive capacity. The link between nutrition and fertility has been widely studied in the scientific field, and there is a lot of evidence in its favor. Healthy eating habits and a lifestyle can greatly enhance one’s ability to conceive. Nonetheless, many people often believe they are following a healthy diet, but most of the time their diet is not suitable for improving fertility. A diet aimed at enhancing procreative capabilities, as well as the physical and mental well-being of patients seeking motherhood, should include foods rich in specific nutrients needed for reproduction and hormonal balance, fetal development, and the health of eggs and sperm [1,2,3]. Despite growing evidence as to the impact of eating habits on human fertility, few studies have examined the public health implications of such an association.

A thorough analysis as to the current scientific evidence on the associations between dietary intake and fertility points to major challenges in the realm of public health. We therefore have set out to review the literature on the relationship between diet and fertility, supported by a retrospective single center cohort study based on our personal experience (IVF Center Villa Sofia Cervello Hospital, Palermo, Italy). This study aligns with currently available research findings and ultimately outlines a set of evidence-based recommendations to address these issues.

A diet relying mostly on unsaturated fats, whole grains, vegetables, and fish has been associated with improved fertility in both women and men. While current evidence on the role of dairy products, alcohol, and caffeine is still inconclusive, saturated fat and sugar have been associated with poorer fertility outcomes in women and men. In addition, obese women and men, i.e., with a body mass index (BMI) ≥ 30 kg/m^2^ [4], have a higher risk of infertility, as do underweight women (BMI < 20 kg/m^2^) [5]. Diet and BMI influence outcomes of clinical infertility treatment. Women who belong to underrepresented minority groups, with lower income and lower education levels overall, have significantly higher rates of infertility than non-Hispanic white women with higher incomes and higher education levels. In light of this, it is worth remarking on the importance of relying on sound nutritional counseling into both clinical guidelines for infertility and national dietary guidelines for people of reproductive age. Further studies on diet, reproductive health, and dietary supplementation can improve our ability to optimize existing fertility programs by providing personalized care to women and men in at-risk groups. Healthy eating habits can greatly contribute to physical and psychological well-being overall, and even increase fertility, in both males and females. Incidentally, infertility can have adverse psychological repercussions as well [6]. A direct consequence is that through diet we can take in the right nutrients needed to support hormonal balance and the health of oocytes and sperm.

## 2. Materials and Methods

The study centered around 70 patients who were enrolled in the pre-treatment period. Patients took two tablets/day for a period of 9 months in the pre-treatment phase. The study is centered around the collection and recording of a series of information and clinical data according to the times and methods described. Ultimately, the duration of supplementation for the couples that could finally be included in the study, in order to standardize the sample without interruption (and therefore drop-out), was 9 months.

The study lasted for a 12-month period and was subdivided as follows:

Pre-phase of monitoring and enrollment of subjects. In this phase, treatment with a multivitamin compound GENANTE, (S&R Farmaceutici S.p.A., Bastia Umbra, Perugia, Italy) had begun. Such a compound is a formulation based on trans-resveratrol supported by a magnesium hydroxide matrix, designed to effectively address the solubility problem of resveratrol, thus substantially improving its dissolution rate, solubility, and, ultimately, bioavailability.

Observed duration period was 270 days during which patients took two tablets each day. Table 1 lays out patient characteristics for each group.

### 2.1. Inclusion Criteria

The study only included infertile women aged 18 to 42 years, with a BMI between 18 and 30 kg/m^2^, with fewer than 3 previous oocyte retrievals, basal FSH < 10 IU/L, number of antral follicles (ranging 2–10 mm in size) > 10, patients diagnosed with “poor ovarian response” (less than 3 oocytes retrieved in previous attempts or who had an estradiol peak at the end of stimulation < 500 pg/mL), and a regular uterine cavity for the expected reproductive function. In addition, patients who underwent hysterosalpingography, hysterography, or hysteroscopy performed no earlier than 12 months after the procedure; had hematological and biochemical parameters within normal limits; and with euthyroidism (with or without treatment) were included in the study. No restrictions regarding the indication of infertility were implemented.

### 2.2. Exclusion Criteria

The following exclusion criteria were applied: primary or secondary ovarian failure, previous episodes of ovarian hyperstimulation syndrome, presence of ovaries inaccessible to oocyte retrieval, persistent ovarian cyst > 20 mm, presence of sactosalpinx, heterologous fertilization, contraindication to pregnancy, atypical genital discharge of unspecified cause, uncontrolled thyroidism, presence of neoplasms, severe alteration of liver or kidney function, or intake of drugs that may interfere with the study.

### 2.3. MAP Intervention Protocol

Patients underwent ovarian stimulation treatment with gonadotropins (recombinant FSH, urinary FSH, FSH + LH) starting from the 2nd–3rd day of the menstrual cycle. The initial dose may vary between 150 and 300 IU, in relation to the ovarian reserve and BMI, as previously assessed. On the 5th day of stimulation, follicular ultrasound monitoring began evaluating the ovarian response. Dosages were adjusted in relation to the outcome of the ultrasound results. In the presence of at least 2 follicles with a diameter of 18 mm, human chorionic gonadotropin was administered for the final induction of ovulation. After 34–36 h, vaginally performed oocyte collection was performed under ultrasound control and local vaginal anesthesia.

Collection of Biological Material: The oocytes were deprived of the cumulus oophorus and subsequently classified by maturity, in accordance with the ESHRE 2012 guidelines [7]. A high-quality oocyte, in metaphase II (MII), was defined as a round oocyte, of normal size, with a regular polar body in the perivitelline space, a homogeneous ooplasm without irregularities, and an adequate thickness of the zona pellucida [7]. These were subsequently fertilized using the IVF/ICSI procedure. Then, 18 h after insemination, fertilization was verified with the formation of pronuclei. Then, the egg was left in culture to verify regular cell replication. All embryos obtained were re-evaluated on the 2nd day, regardless of the day chosen for transfer. The reassessment consists of the consideration of the number of developed blastomeres, the percentage of fragmentation (Grade A: ≤10%; Grade B: >10% to ≤25%; Grade C: >25% to ≤50%; Grade D: >50%), and the appearance of cell division (typical or non-typical), in accordance with consolidated guidelines [8]. The standard ICSI procedure was performed using sperm from the male partner. Embryo transfer was performed between days 3 and 5 after collection of the oocytes, taking into account the characteristics of the patient and the embryo.

Only embryo transfers from fresh cycles were considered. Beta hCG levels were measured to diagnose pregnancy. Clinical pregnancy was established by the presence of a gestational sac, an embryonic pole, and cardiac activity on transvaginal ultrasound, after dosing of beta hCG performed 14 days after the transfer. Finally, luteal phase support was achieved by administering 50 mg/day of intramuscular natural progesterone (ProntogestV R, IBSA, Lugano, Switzerland) or 800 mg/day of micronized progesterone (ProgeffikV R, Effik Italia, Milan, Italy) and maintained until the day of hCG measurement.

## 3. Results

### 3.1. Primary and Secondary Outcomes

**Primary outcomes**

- Effects, intended as biological differences, of GENANTE^®^ treatment on biological parameters deriving from the study of oocyte quantity and quality.

**Secondary outcomes**

- Differences between the two groups in terms of gonadotropin dosage, in the timing of administration/dose adjustments.
- Improvement of the associated reproductive process; i.e., biochemical and/or clinical pregnancy.

### 3.2. Statistical Analysis—Primary Endpoint Analysis

The analysis conducted on the number of mature follicles (>16 mm) highlighted a mean value of 9.4 in the treated group and 5.8 among the controls. The difference of 3.6 (95% CI: from 2.6 to 4.5) was statistically significant (*p* < 0.0001).

Total oocyte count showed a mean value of 9.0 in the treated group and 5.3 in the control group. The difference of 3.7 (95% CI: 2.8 to 4.7) was statistically significant (*p* < 0.0001). The number of M2 oocytes showed a mean value of 7.3 in the treated group and 3.9 in the control group. The difference of 3.4 (95% CI: 2.6 to 4.2) was statistically significant (*p* < 0.0001).

The fertilization rate was 5.7 in the treated group and 2.8 in the control group. The difference of 2.9 (95% CI: 2.3 to 3.6) was statistically significant (*p* < 0.0001). Table 2 outlines a comparison analysis of primary endpoints between treatment and control groups.

### 3.3. Secondary Endpoint Analysis

The analysis of the duration of gonadotropins showed a mean value of 9.3 in the treated group and 9.5 in the control group. The difference of −0.2 (95% CI: −0.36 to −0.04) was statistically significant at the 5% level (*p* < 0.05). The analysis of gonadotropin dosage showed a mean value of 191.8 in the treated group and 234.6 among the controls. The difference of −42.9 (95% CI: from −65.4 to −20.4) was statistically significant (*p* < 0.001).

The number of frozen blastocysts showed a mean value of 3.4 in the treated group and 1.2 in the control group. The difference of 2.2 (95% CI: 1.8 to 2.7) was statistically significant (*p* < 0.0001). Table 3 lays out a comparative analysis of secondary endpoints between treatment and control groups.

Primary endpoints: The study group showed higher numbers of mature follicles, total oocytes retrieved, and M2 oocytes retrieved compared to controls. The study group also showed a higher fertilization rate as well as higher numbers of cleavage embryos per patient, blastocysts per patient, and frozen blastocysts.

Secondary endpoints: The study group was administered a lower total amount of gonadotropins during stimulation compared to the control group.

The time of administration of gonadotropins, necessary to reach follicle maturity, was shorter in patients belonging to the study group compared to controls; it can also be observed that patients in the study group underwent fewer adjustments to the initial dosage of gonadotropins (less frequent adjustments of the “starting dose”), compared to controls. No significant differences in biochemical or clinical pregnancy rates were found between the two groups.

## 4. Discussion

The World Health Organization (WHO) considers the absence of conception after 12/24 months of regular unprotected sexual intercourse as a pathological condition [9]. In such cases, infertility is diagnosed, which can be male, female, or couple-related infertility. In Italy, the infertility rate is roughly 15%, compared to a world average that ranges from 10 to 12%. Although infertility is often associated with women, male physiological factors reportedly account for ~25% of cases, which highlights the need to focus on both partners [10,11]. Thus, it is now quite common to use the phrase “couple infertility”. Infertility rates have remained high despite the increase in the use of assisted reproductive technologies (ART) in recent years [12]. For this reason, research has long sought to identify modifiable risk factors for infertility, and nutritional factors have long been investigated. Evidence points out that diet and nutritional supplementation with nutraceuticals may play an important role in modifying fertility-related outcomes in both men and women. The link between diet and fertility has been extensively researched over the years. A research study published in 2007 [13] has had a strong impact on subsequent examinations. In this study, 17,544 women with no history of infertility were followed for 8 years while they were trying to achieve pregnancy.

The study found that a tailored “fertility diet” model was associated with a lower risk of infertility due to an ovulatory disorder. The proposed diet included a higher consumption of monounsaturated fats rather than trans fats; plant-based rather than animal-based protein sources; low-glycemic carbohydrates; high-fat dairy products; and multivitamins and iron from plants and supplements.

A combination of five or more low-risk lifestyle factors, including diet, weight control, and physical activity, was found to be associated with a 69% lower risk, although the impact of rigorous dieting and exercising is still being investigated in terms of its potential benefits and contraindications as well [14].

The result of the study therefore demonstrated that a “fertility diet” model can favorably influence fertility in healthy women, as well as prevent most cases of infertility due to ovulation disorders. Particularly significant in that regard are the studies conducted by Erasmus MC-University Medical Center Rotterdam [15] and the one published by the *Journal of Nutritional & Environmental Medicine* [16], both of which found eating habits and healthy nutrition to be crucial factors in both female and male fertility. The access to in vitro fertilization (IVF) techniques for women at an increasingly advanced age is due to the tendency in our society to try to have children at an increasingly advanced age, when fertility is reduced. In fact, the reproductive capacity of the couple declines with age. Aging reportedly has a role in mitochondrial dysfunction [17]. Energy production through the mitochondrial respiratory chain is necessary in various phases of female gametogenesis [18].

During oocyte maturation, the number and functionality of mitochondria rapidly increase to prepare for the energy expenditure associated with early embryonic development [19]. Many fertility-related parameters are connected with mitochondrial metabolic status [20]. Some natural substances can effectively modulate mitochondrial activity. For instance, resveratrol has been reported to modify mitochondrial activity at various levels: transcriptional, through the expression of the SIRT family (sirtuin NAD-dependent deacetylase); functional, through the modulation of the respiratory chain; and epigenetic [20]. All such mechanisms positively affect resistance to apoptosis and result in greater energy availability [19].

Trans-resveratrol is poorly bioavailable due to reduced absorption mainly related to its low solubility and rapid metabolism characterized by its conversion to glucuronidated and sulfated compounds, which are generally inactive and with high renal clearance [21]. According to the Biopharmaceutical Classification System (BCS) [22], resveratrol belongs to class 2. It is part of those molecules characterized by low water solubility (about 30 mg/L), but with high membrane permeability (logP~3.1) [23]. In humans, following oral administration, resveratrol is detected in plasma after about half an hour, indicating that its absorption begins already at gastric level and reaches a peak plasma concentration sub-micromolar. Recently, a formulation named REVIFAST (Vemax Pharma, Belgrade, Serbia) has been developed based on the interaction of trans-resveratrol with magnesium hydroxide which, by modifying the gastric dissociation profile, increases its bioavailability. REVIFAST is able to solubilize faster and in larger quantities than trans-resveratrol alone, with significant advantages in biopharmaceutical terms. From a physical point of view, REVIFAST is a solid dispersion, i.e., a new formulation in which the active ingredient relies on support by high-safety inorganic material (magnesium hydroxide), which improves its performance without any chemical alteration in the structure of the natural product, thus making it possible to fully harness the pharmacological properties of this molecule.

Vitamin B12 (cyanocobalamin) is necessary for the metabolism of odd-chain fatty acids and therefore necessary for energy metabolism and cell proliferation. Supplementation with folic acid and vitamin B-12 has been associated with positive reproductive outcomes in both natural pregnancies and those following treatment with assisted reproductive technology (ART) [24], and preliminary data show benefits even in terms of follicular output rate (FORT) in patients who took multinutrient supplementation (omega-3, coenzyme Q10, folic acid, selenium, vitamin E, and catechins) [25]. Particularly, vitamin B6 (pyridoxine) affects various biochemical reactions involving both the metabolism of amino acids and that of various other compounds. Higher levels of vitamin B6 have been found in fertile women compared to infertile women [26]. Vitamin D3 is well known for its role in maintaining calcium homeostasis and promoting bone mineralization. Growing evidence points to a correlation between vitamin D deficiency and decreased female fertility [27]. A 2024 study [28] shows how children of women with low levels of vitamin D during pregnancy were characterized by increased activity of the reproductive axis and faster postnatal growth of the genital organs. Minipuberty is a term that refers to the temporary and sex-specific activation of the hypothalamic–pituitary–gonadal axis, which is involved in the development of male and female genital organs. The study, the first of its kind, has taken into account minipuberty in the offspring of vitamin D-deficient women. Three matched groups of girls were included in the study population: those born to women with vitamin D deficiency (25OHD concentration less than 50 nmol/L), daughters of women with vitamin D insufficiency (25OHD concentration between 50 and 75 nmol/L), and daughters of healthy females (25OHD concentration between 75 and 150 nmol/L). Salivary concentrations of estradiol, progesterone, 17-hydroxyprogesterone, and androgens, as well as urinary concentrations of FSH and LH, were analyzed during the first 18 months of life (once a month in the first 6 months, bimonthly between months 6 and 12, and every three months thereafter). The size of the reproductive organs, ovaries, uterus, and breasts was assessed at every subsequent examination. In daughters of mothers with vitamin D deficiency, FSH, LH, and estradiol concentrations were higher and detectable for a longer period of time, while ovarian volume, uterine length, and breast diameter were found to be larger than in the other groups. Within the same detection period, females born to women with vitamin D deficiency had higher FSH levels than the daughters of healthy women, and such groups also showed different breast diameter. Such findings point to a low vitamin D status during gestation as a factor determining more pronounced and long-lasting activation of the reproductive axis and are also associated with an increase in the size of sexual organs, the extent of which is linked to vitamin D deficiency levels. The role of vitamin D deficiency in metabolic disorders and infertility in women with polycystic ovary syndrome (PCOS) has been supported by recent research data [29,30]. Both insulin resistance (IR) and endometrial receptivity seem to be favorably affected by vitamin D supplementation. On the other hand, excessively high levels of vitamin D seem to have a detrimental role on oocyte development and embryo quality. Therefore, we encourage low-dose supplementation (400–800 IU/day) especially in women with vitamin D deficiency who have metabolic disorders such as PCOS. Regarding reproductive health, we recommend vitamin D supplementation in selected populations, only during specific times of the ovarian cycle, to support the luteal phase. However, currently available clinical studies published so far are still somewhat inconclusive as to supplementation dosage and timing and further studies are needed to corroborate such correlations [31].

In women with a history of endometriosis, vitamin C and vitamin E supplementation has been linked to improved fertility rates. A 2021 study [32] enrolled 60 women of reproductive age (15–45 years) with pelvic pain in this triple-blind clinical trial. They had stages 1–3 of laparoscopically confirmed endometriosis. Participants were randomized to either group A (*n* = 30), which was administered the combination of vitamin C (1000 mg/day, 2 tablets of 500 mg each) and vitamin E (800 IU/day, 2 tablets of 400 IU each), or group B (*n* = 30), which was administered placebo pills daily for 8 weeks. After treatment with vitamin C and vitamin E, a significant reduction in MDA and ROS was found compared to the placebo group. No significant decline was found in total antioxidant capacity after treatment. However, the severity of pelvic pain (*p*-value < 0.001), dysmenorrhea (*p*-value < 0.001), and dyspareunia (*p*-value < 0.001) significantly decreased in the treatment group after 8 weeks of supplementation. By virtue of their antioxidant capacity, vitamin C and E can “turn off” endometriosis, thus improving fertility prognosis in women undergoing treatment. Another recent study funded by the Division of Reproductive Endocrinology and Infertility at the University of North Carolina at Chapel Hill, evaluated the usefulness of omega-3 administration in women for the purpose of preserving fertility [33]. The study, which was not a randomized controlled trial, pointed to women who used omega-3 supplements as a more health-conscious population. We attempted to address this issue by adjusting our model for several factors. Additionally, the omega-3 fatty acid supplements used by TTC participants included several types and brands with varying dosages of omega-3 fatty acids. Women reported the type of supplement they were taking, but not the concentration of omega-3 in that supplement. Therefore, no comparison can be drawn between the dosage or a dose-response relationship in this study. However, omega-3 supplementation may represent a feasible and inexpensive modifiable factor for improving fertility. Randomized controlled trials are needed to further investigate the benefits of omega-3 supplementation for women seeking natural conception.

Folate plays an essential role in regulating amino acids and nucleic acids at the metabolic level and in one-carbon metabolism. Folate must be obtained from the diet, and supplementation is strongly recommended in populations at risk of deficiency due to of specific conditions. Folic acid is the synthetic form of the vitamin, usually incorporated into foods and supplements. In the body, it must be reduced to the bioactive folate derivative (6S)5-MTHF by cellular metabolism. Folate deficiency is linked to many health problems, such as neurological disorders, and may increase the risk of cardiovascular disease [34]. Patients of childbearing age and pregnant women, in addition to women with MTHFR polymorphism, exhibit the highest rates of folate deficiency. Folate supplementation is substantially helpful for the inhibition of neural tube defect (NTD) in pregnancy and for lowering homocysteine levels. Moreover, (6S)5-MTHF supplementation in pregnancy is advisable instead of folic acid, thanks to its ability to bypass the polymorphic enzyme blockade of folic acid metabolism. The use of (6S)5-MTHF can also mitigate the risk of deleterious effects of unmetabolized folic acid (UMFA) associated with the use of a supraphysiological dose of folic acid [34].

Methylation regulates numerous biochemical/physiological stages, and the importance of folates and one-carbon cycles for proper methylation has frequently been overlooked. Adding FA through nutrition has proved useful to lower NTD rates during pregnancy. However, the generation of circulating UMFA is now reason for concern, since it can compete heavily with the natural “active” folate metabolite, 5-MTHF, particularly in neonates. When assessing physiological effects by measuring serum folate values, fluorescence-based assays gauge UMFA along with other folate molecules, which can lead to results and misleading conclusions. Most IVF (in vitro fertilization) units recommend the intake of FA supplements before ART cycles, even in in high doses (5 to 15 mg) at times. It is therefore essential to take into account the possible impact of UMFA on germ cells in the long run. UMFA-induced sperm alterations in animal sperm have also been observed, and such alterations appear likely to occur in humans as well. Differential sex-related abnormalities are clearly linked to varying degrees of gradual resetting of epigenetic marks between males and females [35,36,37,38,39]. It is worth focusing on how germ cells may be impacted, since the consequences/effects may not be easily perceived [40,41,42,43]. The effects on gametogenesis will only become apparent in the next generation, after reproduction of the offspring, about 25 years later. Furthermore, depending on the sex, epigenetic abnormalities can skip a generation and then resurface.

Synthetic FA can be an issue for MTHFR SNP carriers (especially for newborns) from a biochemical and physiological standpoint, since the initial phases of FA metabolism are already compromised. 5-MTHF can get over the initial strict metabolic phase and does not result in the accumulation of unmetabolized compounds; still, it is advisable to stich to a physiological dose of approximately 500 µg/day, which may be raised during the first trimester of pregnancy. In couples presenting “ idiopathic infertility”, the paternal effect should not be overlooked [44,45]. Routine testing of both partners for MTHFR SNPs is indicated, as treatment with 5-MTHFR may be substantially useful.

A 2022 randomized, double-blind, controlled study [46] has corroborated the potential value of myo-inositol and melatonin supplementation in PCOS patients undergoing IVF cycles. Polycystic ovary syndrome (PCOS) can bring about anovulation in women of reproductive age and is one of the pathological factors involved in IVF failure. In fact, patients with PCOS generally have poor quality oocytes, hence the need to rely on treatment aimed at improving the oocyte quality for such patients. Myo-inositol [47] and melatonin have been shown to be effective predictors of positive IVF outcomes, correlating with high oocyte quality. A 2022 review article [48] also points out that dietary supplementation of melatonin and vitamin D, by reducing oxidative stress and turning off chronic inflammation, could have a favorable effect on fertility, ensuring good reproductive function. Melatonin is an interesting compound, as is vitamin D, which has a pleiotropic activity and responds to light–dark cycles. From a scientific perspective, melatonin has an antioxidant value action capable of crossing the blood–brain barrier, tackling inflammation and interacting with the intestinal microbiome. Thus, a melatonin imbalance can signal a “dark deficiency”, just as vitamin D levels can indicate whether or not a person has a “light deficiency”. Doctors should therefore provide both nutritional and lifestyle recommendations to optimize melatonin intake for their patients.

A recent 2024 study [49] has pointed to a lower expression of melatonin receptors, in addition to reduced serotonin synthesis, as significant factors in “ovarian aging”. The chief melatonin receptors that were accounted for are the MT1 and MT2 types. The study centered around the expression of genes and synthesis of MT1 and MT receptors, in addition to serotonin synthesis in the ovaries during ontogenesis. Histological materials from the ovaries of newborns; women of young and advanced reproductive age; and premenopausal, menopausal, and postmenopausal patients were analyzed. RT-PCR and immunohistochemistry were used to analyze MTI and MT receptors and serotonin expression and synthesis. Both serotonin synthesis, and MTI and MT2 receptors in the ovaries, have been found to significantly decrease during ontogenesis, with the sharpest decrease being reported in samples obtained from one-year-old infants, pubescent girls, and menopausal women. Moreover, a considerable 2.3- to 7.6-fold dip in the expression of the MTNRIA and MTNR1B genes in the ovaries has been reported in one-year-old infants, adolescents, and middle-aged women. Such data are highly valuable in order to gain a deeper understanding of the fundamental mechanisms through which aging affects the female reproductive system, and to further our search for molecules that predict aging, thus preserving fertility.

The value of nutrition plans as major factors for infertility patients has been supported by clinical guidelines, hence their relevance is clinical as well as medicolegal [50,51]. As a matter of fact, any therapeutic pathway aimed at restoring fertility or implementing a MAP plan enabling patients to achieve parenthood must be deeply rooted in evidence-based guidelines, best practices, and recommendations in order to be sound from a medicolegal standpoint [52]. Nutritional counseling is therefore of utmost importance in order to maximize the possibilities of successfully treating infertility. As recent findings published in the *Journal of the American Medical Association* show, a lower likelihood of pregnancy loss has been linked to pre-conception healthy dietary patterns before infertility treatment [53]. In light of such highly significant correlation, any failure to provide counseling as to such requirements could contribute to unfavorable outcomes and even result in litigation and negligence-based malpractice, especially ones possibly associated with poor or lacking nutrition [54,55]. From that perspective, dietary/nutritional counseling ought to be prioritized for patients with infertility to the same extent that other forms of reproductive counseling are implemented [56,57], particularly in light of the toll rising obesity rates are taking on fertility [58,59,60]. Against the backdrop of the post-COVID-19 pandemic era, it is worth mentioning the destructive impact of the pandemic on both fertility and infertility treatments [60,61,62,63,64]. Nutritional counseling should therefore be acknowledged as a key tool in making up for the losses brought about by the pandemic [65,66], and any such intervention needs to be thoroughly documented and provable, should litigation arise from adverse outcomes. It is in fact worth bearing in mind that particularly under civil statutes, the onus is placed on the doctors and facilities to prove compliance with evidence-based guidelines and recommendations [54,67,68]. Nutritional counseling is in fact a process with unique complexities [69], hence it needs to rely on highly trained professionals with a solid grounding in medical/clinical as well as psychological and behavioral sciences (e.g., evaluating and adapting to each patient’s motivation and awareness/knowledge levels, seeking involvement from patients and maintaining such an involvement, and varying between counseling formats to best suit the profile of all involved) for each patient’s needs to be met as effectively and beneficially as possible [69,70,71]. It is therefore worth encouraging the framing of more comprehensive, evidence-based global and regional weight control guidelines, in addition to including nutritional counselling indicators and criteria as an integral part of quality-of-care frameworks and blueprints both at the antenatal care and postnatal care levels.

### Study Strengths and Weaknesses

The chief strength of this study is its focus on supplementation with a nutraceutical that has contributed to increasing reproductive outcomes, without significant side effects. This aspect is still somewhat under-researched and takes on even greater relevance in light of lower and lower birth rates, in Western countries especially. The main limitation has to do with data selection bias, which can influence the representativeness of the data with confounding and causality factors. In such respects, retrospective studies are usually particularly vulnerable because of the difficulties in controlling all the variables. This is in contrast to prospective studies, where a more rigorous analysis of the variables themselves can be framed and better control of the confounding effects can be obtained. In retrospective studies, such a range of control is limited. The outcomes can therefore be measured and assessed in a non-uniform way, which may result in substantial differences in the conclusions.

## 5. Conclusions

Pre-treatment adherence to a fertility-friendly diet has been linked to better chances of achieving a pregnancy, and even live birth, in patients undergoing ART. A 9-month period of dietary supplementation with a resveratrol-based multivitamin nutraceutical leads to better biological effects on ICSI cycles. In light of the worrisome declining fertility rates in Western countries (which are in large part influenced by socioeconomic factors beyond the scope of this manuscript), it is essential to maximize the opportunity to achieve pregnancy and parenthood for those who want to start a family. From that perspective, nutritional counseling is to be deemed an extremely valuable component of a multidisciplinary effort, aimed at providing strictly tailored personalized strategies to overcome infertility whenever possible. Such an undertaking needs to be deeply rooted in evidence-based guidelines and validated recommendations, so as to ensure the best therapeutic pathway as well as medicolegal viability of any such intervention.

## Figures and Tables

**Table 1:** Patient characteristics between treatment and control groups.

Patient Characteristics	Treatment Group	Control Group	*p*-Value
Age (mean)	31.5	30.5	n.s.
BMI (mean)	24.6	24.9	n.s.
AMH (mean)	1.4	1.4	n.s.

**Table 2:** Comparison analysis of primary endpoints between treatment and control groups.

				95% CI		*p*-Value
Primary Endpoints	Treatment Group (Mean Value)	Control Group (Mean Value)	Difference in Mean Values	Min	Max	
Number of mature follicles	9.4	5.8	3.6	2.6	4.5	<0.0001
Total oocyte count	9.0	5.3	3.7	2.8	4.7	<0.0001
Number of M2 oocytes	7.3	3.9	3.4	2.6	4.2	<0.0001
Fertilization rate	5.7	2.8	2.9	2.3	3.6	<0.0001

**Table 3:** Comparative analysis of secondary endpoints between treatment and control groups.

				95% CI		*p*-Value
Secondary Endpoints	Treatment Group (Mean Value)	Control Group (Mean Value)	Difference in Mean Values	Min	Max	
Duration of gonadotropins	9.3	9.5	−0.20	−0.36	−0.04	<0.05
Gonadotropin dosage	191.8	234.6	−42.9	−65.4	−20.4	<0.001
Starting dose	174.6	198.8	−24.1	−49.4	1.1	n.s.
Transferred blastocysts	1.4	1.2	0.17	−0.001	0.34	0.05
Frozen blastocysts	3.4	1.2	2.2	1.8	2.7	<0.0001
Beta hCG	0.34	0.31	0.03	−0.13	0.19	n.s.

## Data Availability

All data are available upon request from the corresponding author.
